# Supplementary material for: From sequence to enzyme mechanism using multi-label machine learning
Source: BMC Bioinformatics. 2014 May 19;15:150. doi: 10.1186/1471-2105-15-150 (PMC4229970; doi:10.1186/1471-2105-15-150)
Supplement: Additional file 2 — Java code of ml2db. Additional file ml2db_code.tar.gz contains the Java source code to run the multi-label machine learning experiments and save the results to database. The code’s Javadoc is included. [file 1471-2105-15-150-S2.zip › additional file 2/ml2db/ml2db/doc/mulan/evaluation/loss/ErrorSetSize.html]

ErrorSetSize


JavaScript is disabled on your browser.


- Overview
- Package
- Class
- Use
- Tree
- Deprecated
- Index
- Help

- Prev Class
- Next Class

- Frames
- No Frames

- All Classes

- Summary:
- Nested |
- Field |
- Constr |
- Method

- Detail:
- Field |
- Constr |
- Method


mulan.evaluation.loss

## Class ErrorSetSize

- java.lang.Object
- - mulan.evaluation.loss.RankingLossFunctionBase
  - - mulan.evaluation.loss.ErrorSetSize

- All Implemented Interfaces:
  :   java.io.Serializable, MultiLabelLossFunction, RankingLossFunction

  Direct Known Subclasses:
  :   RankingLoss

  ---

    

  ```
  public class ErrorSetSize
  extends RankingLossFunctionBase
  ```

  Implementation of the ErrorSetSize loss function, which computes the size of
  the error set. The error set is composed of all possible label pairs,
  where one is relevant and the other is not, and which satisfy the condition
  that the relevant label is ranked lower than the irrelevant one.

  Version:
  :   2010.11.10

  Author:
  :   Grigorios Tsoumakas

  See Also:
  :   Serialized Form

- - ### Constructor Summary

    Constructors

    Constructor and Description

    `ErrorSetSize()`
  - ### Method Summary

    Methods

    | Modifier and Type | Method and Description |
    | --- | --- |
    | `double` | `computeLoss(int[] ranking, boolean[] groundTruth)` Computes the ranking loss function |
    | `java.lang.String` | `getName()` Returns the name of the loss function |

    - ### Methods inherited from class mulan.evaluation.loss.RankingLossFunctionBase

      `computeLoss`
    - ### Methods inherited from class java.lang.Object

      `equals, getClass, hashCode, notify, notifyAll, toString, wait, wait, wait`

- - ### Constructor Detail


    - #### ErrorSetSize

      ```
      public ErrorSetSize()
      ```
  - ### Method Detail


    - #### getName

      ```
      public java.lang.String getName()
      ```

      **Description copied from interface: `MultiLabelLossFunction`**

      Returns the name of the loss function

      Returns:
      :   the name of the loss function


    - #### computeLoss

      ```
      public double computeLoss(int[] ranking,
                       boolean[] groundTruth)
      ```

      **Description copied from interface: `RankingLossFunction`**

      Computes the ranking loss function

      **Specified by:**
      :   `computeLoss` in interface `RankingLossFunction`

      **Specified by:**
      :   `computeLoss` in class `RankingLossFunctionBase`

      Parameters:
      :   `ranking` - the ranking of the learner for an example
      :   `groundTruth` - the ground truth of the example

      Returns:
      :   the value of the loss function


- Overview
- Package
- Class
- Use
- Tree
- Deprecated
- Index
- Help

- Prev Class
- Next Class

- Frames
- No Frames

- All Classes

- Summary:
- Nested |
- Field |
- Constr |
- Method

- Detail:
- Field |
- Constr |
- Method
